# Frequency domain maximum correntropy criterion spline adaptive filtering

**DOI:** 10.1038/s41598-021-01863-6

**Published:** 2021-12-15

**Authors:** Wenyan Guo, Yongfeng Zhi, Kai Feng

**Affiliations:** 1 School of Automation, Northwestern Polytechnical University, Xi’an, 710129 China; 2 Science and Technology on Electromechanical Dynamic Control Laboratory, Xi’an, 710065 China; 3 Science and Technology on Electro-Optical Information Security Control Laboratory, Tianjin, 300308 China

**Keywords:** Applied mathematics, Electrical and electronic engineering

## Abstract

A filtering algorithm based on frequency domain spline type, frequency domain spline adaptive filters (FDSAF), effectively reducing the computational complexity of the filter. However, the FDSAF algorithm is unable to suppress non-Gaussian impulsive noises. To suppression non-Gaussian impulsive noises along with having comparable operation time, a maximum correntropy criterion (MCC) based frequency domain spline adaptive filter called frequency domain maximum correntropy criterion spline adaptive filter (FDSAF-MCC) is developed in this paper. Further, the bound on learning rate for convergence of the proposed algorithm is also studied. And through experimental simulations verify the effectiveness of the proposed algorithm in suppressing non-Gaussian impulsive noises. Compared with the existing frequency domain spline adaptive filter, the proposed algorithm has better performance.

## Introduction

In dedicated electronic circuits due to the inherent nonlinearity of certain hardware components, nonlinear modeling and system identification are important. The power amplifier (PA), for example, is typically operated in its nonlinear region to improve power efficiency. The nonlinear spline adaptive filter (SAF) algorithm is widely utilized in nonlinear modeling due to its simple structure. The basic framework of SAF includes Wiener spline filter^1^, Hammerstein spline filter^2^, Sandwich 1 SAF, Sandwich 2 SAF^3^. Some researchers carry out the steady-state performance analysis study of SAF^4,5^. Some researchers have improved the SAF algorithm from the cost function on the basis of Wiener spline filter. A kind of normalized SAF algorithm (SAF-NLMS), is improved the stability of SAF, proposed by Guan^6^. A sign normalized least mean square algorithm (SNLMS) based on SAF, and the variable step-size scheme is introduced, called SAF-VSS-SNLMS, proposed by Liu^7^. The algorithm through introduce momentum in stochastic gradient descent, formed SAF-ARC-MMSGD^8^, against an impulsive environment. The weight update of the normalized subband spline adaptive filter algorithm is conducted the principle of minimum disturbance^9^. The maximum versoria criterion (MVC) is introduced nonlinear spline adaptive filter, formed SAF-MVC-VMSGD^10^. The algorithms combine the logarithmic hyperbolic cosine (LHC) as cost function for nonlinear system identification^11,12^. Based on logarithmic hyperbolic cosine (LHC) cost function, proposed novel cost function exponential hyperbolic cosine function (EHCF)^13^, generalized hyperbolic secant^14^, formed adaptive filtering. The above SAF type algorithms are carried out in time domain. As the order of the filter increases, the computational complexity will increases. In order to solve this problem, a frequency domain spline adaptive filter (FDSAF) is proposed^15^. Frequency domain spline adaptive filtering (FDSAF) can effectively reduce the computational complexity.

However, the frequency domain spline adaptive filtering is derived by minimising the squared value of the instantaneous error, unable to suppress non-Gaussian impulsive noises. According to the maximum correlation entropy criterion (MCC) combined with adaptive filtering^14,16–18^, combined with spline adaptive filtering^19–21^, the robustness of MCC is demonstrated. To suppression non-Gaussian impulsive noises along with having comparable operation time, a frequency domain maximum correntropy criterion spline adaptive filter (FDSAF-MCC) is developed in this paper.

## Results

Several experiments are implemented in order to verify the performance of the proposed FDSAF-MCC against non-Gaussian environments. The algorithm performance is measured by the mean square error (MSE), $\rm{MSE} = 10 \log_{10} {[e(k)]^2}$.

**Figure 1:**
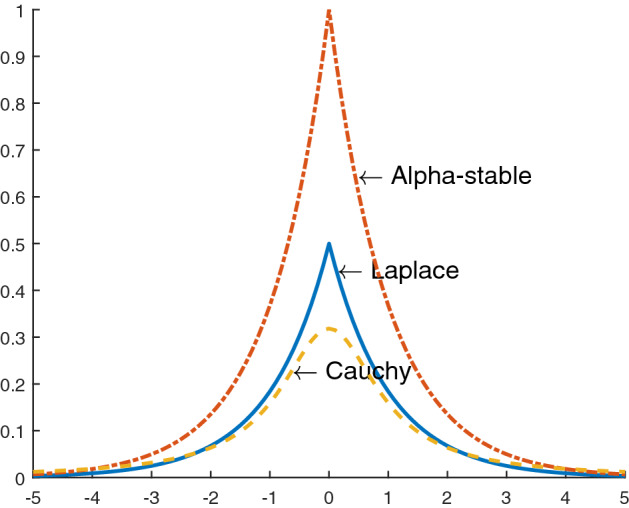
Comparison of the probability density function of heavy tail noise.

Non-Gaussian noise models are usually classified into heavy-tailed non-Gaussian noise (e.g., Alpha-stable, Laplace, Cauchy, etc.) and light-tailed non-Gaussian noise (i.e., binary, uniform, etc.). This paper mainly focuses on heavy-tail noise. The comparison of the probability density function of heavy tail noise as shown in Fig. 1. We can know that compared with other noise models, alpha-stable distribution noise has heavier tails and sharp peaks. Therefore, an alpha-stable distribution is uitilized for the experiment. An alpha-stable distribution, with $\alpha \in \left( 0,2\right]$ is a characteristic exponent representing the stability index which determines the strength of impulse, $\beta \in \left[ -1,1 \right]$ is a symmetry parameter, $\iota >0$ is a dispersion parameter, and $\varrho$ is a location parameter^22^. Which can be expressed as

1 $$\begin{aligned} \begin{aligned} f(t)&= \exp {\left\{ j\varrho t - \iota {\left| t \right| }^\alpha {\left[ 1 + j \beta sign(t) S(t,\alpha ) \right] } \right\} } \end{aligned} \end{aligned}$$

Where

2 $$\begin{aligned} \begin{aligned} S(t,\alpha )&= \left\{ \begin{array}{ll} \tan {\left( \frac{\alpha \pi }{2} \right) }, &{} \alpha \ne 1 \\ \left( \frac{2}{\pi } \right) \log \left| t \right| , &{} \alpha =1 \end{array} \right. \end{aligned} \end{aligned}$$

In this paper, the parameters of alpha-stable distribution are set as follows, $\alpha =1.6, \beta = 0, \iota = 0.05, \varrho = 0$. We adding to the output of the unknown system, an independent white Gaussian noise *v*(*n*) with the signal to noise ratio (SNR=30dB). In adaptive system, the frequency domain weight is initialized as ${\mathbf {w}}_F(0)=FFT [1, 0, . . ., 0]^T \in {\mathbb {R}}^{2M\times 1}$. The spline knots are initialized as ${\mathbf {G}}(0)= [-2.2, -2.0, \ldots , 2.0, 2.2]^T$, with the interval $\Delta x = 0.2$. The input signal *x*(*n*) is a stochastic process with uniform distribution limited to $[-1, 1]$, the signal samples number are 100,000. The MSE curves are obtained through independent Mento Carlo trials. The parameters of the experiments are set as Table 1.

**Table 1:** Parameters of three kinds experiments.

Experiment	Parameters
1	$M=100, \mu _w=0.001, \mu _g=0.001, trials = 100$
2	$M=1000, \mu _w=0.0005, \mu _g=0.01, trials = 200$
3	$M=100, \mu _w=0.001, \mu _g=0.001, trials = 100$

### Experiment 1

 Experiment 1, the nonlinear system to be identified is composed by a linear FIR filter followed by a nonlinear spline curve. The linear FIR filter system transfer function is described

3 $$\begin{aligned} \begin{aligned} L(z)&=\frac{0.1032z^{-1}-0.0197z^{-2}-0.0934z^{-3}}{1-2.628z^{-1}+2.3z^{-2}-0.6703z^{-3}} \end{aligned} \end{aligned}$$

The control points of nonlinear spline curve are setting as ${\mathbf {g}}^* = [-2.2, -2.0,$ $\ldots$, $-0.8, -0.91, 0.42, -0.01, -0.1, 0.1, -0.15, 0.58, 1.2, 1.0, 1.2$,$\ldots$, $2.0, 2.2]^T$^23^.

**Figure 2:**
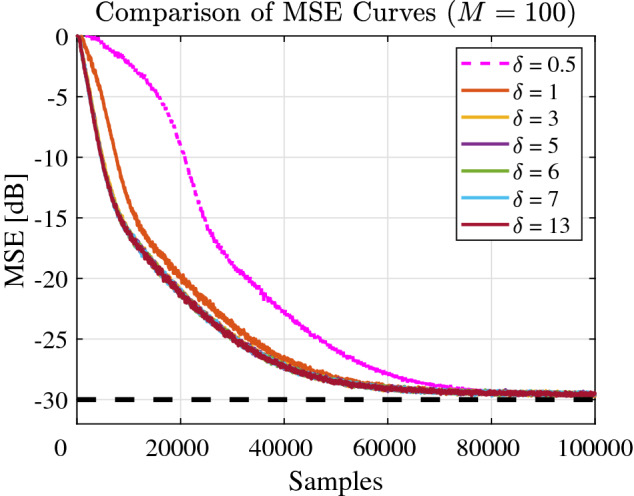
MSE curves of FDSAF-MCC different $\delta$ parameters.

Figure 2 shown the MSE curves in different $\delta$ parameters. This work is carried out in without non-Gaussian noise environment. With the parameter $\delta$ increase, the convergence performance of MSE curve becomes better. However, when the parameters $\delta$ increase to a certain value, the MSE curve will not change any more. In subsequent experiments, setting the parameter $\delta =6$.

**Figure 3:**
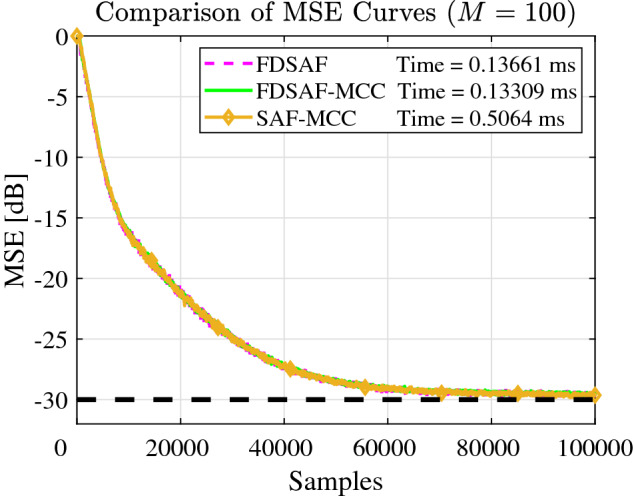
Experiment 1: Comparison of MSE curves of FDSAF, FDSAF-MCC, and SAF-MCC algorithms without non-Gaussian noise.

Figure 3 shown the MSE curves comparison between FDSAF, the proposed FDSAF-MCC, and SAF-MCC under without non-Gaussian noise environment. The convergence performance of the three algorithms is consistent, but the running time of FDSAF-MCC is 0.13309 ms, which is shorter, than the FDSAF, which is 0.13661 ms, than the SAF-MCC, which is 0.5064 ms.

**Figure 4:**
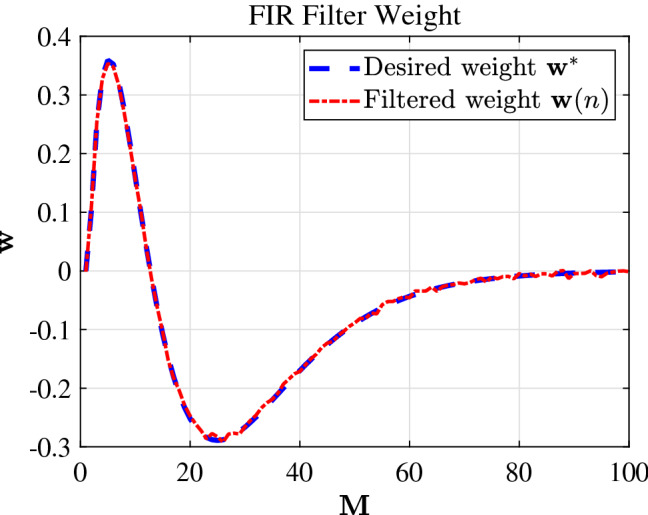
FIR filter weight of FDSAF-MCC under non-Gaussian noise.

**Figure 5:**
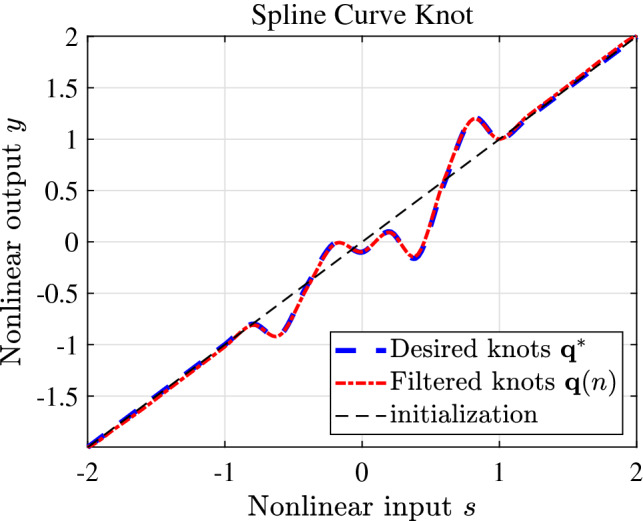
Spline knots of FDSAF-MCC under non-Gaussian noise.

The FDSAF-MCC algorithm to track the weight of FIR filtering with non-Gaussian noise environment, as shown in Fig. 4. The proposed algorithm has good tracking performance. The FDSAF-MCC algorithm to track the spline knots with non-Gaussian noise environment, as shown in Fig. 5. The proposed algorithm has good tracking performance.

**Figure 6:**
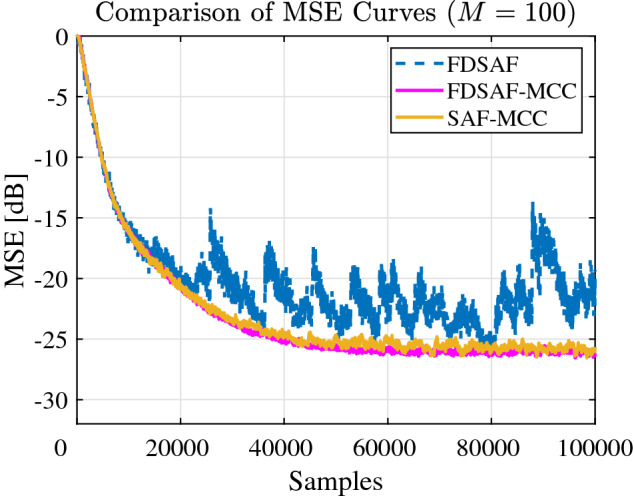
Experiment 1: Comparison of MSE curves of FDSAF, FDSAF-MCC, and SAF-MCC algorithms with impulsive noise.

Figure 6 shown the MSE curves comparison between FDSAF, the proposed FDSAF-MCC, and SAF-MCC under non-Gaussian noise environment. The convergence performance of FDSAF algorithm is bad, the MSE curve oscillates randomly. The proposed FDSAF-MCC and SAF-MCC algorithms have better convergence performance.

**Figure 7:**
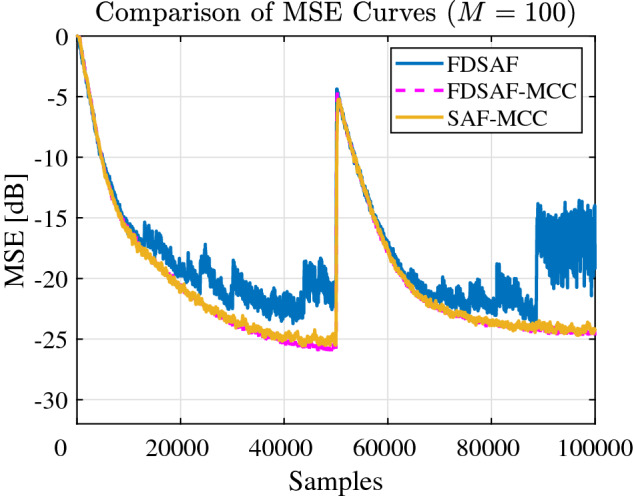
Experiment 1: Comparison of MSE curves of FDSAF, FDSAF-MCC, and SAF-MCC algorithms under impulsive noise and a sudden change.

Figure 7 shown the MSE curves comparison between FDSAF, the proposed FDSAF-MCC, and SAF-MCC under non-Gaussian noise and a sudden change. The proposed FDSAF-MCC and SAF-MCC algorithms have better convergence performance. After a sudden change, the MSE curve converges of the FDSAF-MCC and the SAF-MCC algorithms, the MSE curve divergent of the FDSAF algorithm.

### Experiment 2

 Experiment 2, consists of a 7-order linear FIR filtering sub-system and a saturated nonlinear sub-system. The linear subsystem with 7th order FIR filtering is shown

4 $$\begin{aligned} \begin{aligned} L(s)&=\frac{-2.8e12s^3+4.6e18s^2+6.4e21s+3.2e27}{s^7+1e4s^6+2.6e9s^5+1.2e13s^4+1.2e18s^3+2.1e21s^2+9.4e23s+9.7e26} \end{aligned} \end{aligned}$$

The saturated nonlinearity is described by

5 $$\begin{aligned} f(p)=arctan(2p) \end{aligned}$$

*p* described the nonlinear input, *f*(*p*) described the nonlinear output.

**Figure 8:**
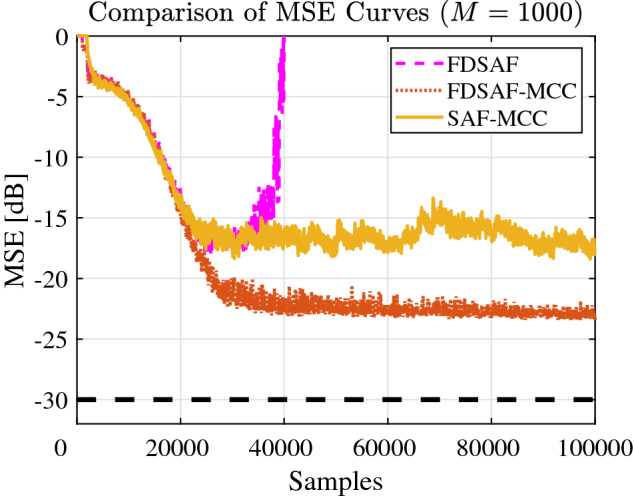
Experiment 2: MSE curves of FDSAF, FDSAF-MCC, and SAF-MCC under impulsive noise.

Figure 8 shown the MSE curves comparison between FDSAF, the proposed FDSAF-MCC, and SAF-MCC under impulsive noise environment. The convergence performance of FDSAF algorithm is poor. FDSAF algorithm cannot suppress impulsive noise. The proposed FDSAF-MCC algorithm has the better convergence performance than SAF-MCC algorithm.

**Figure 9:**
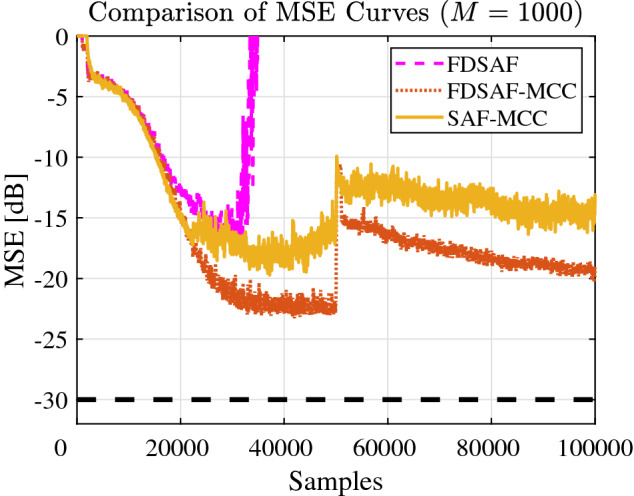
Experiment 2: MSE curves of FDSAF, FDSAF-MCC, and SAF-MCC under a sudden change.

Figure 9 shown the MSE curves comparison between FDSAF, the proposed FDSAF-MCC, and SAF-MCC under impulsive noise and a sudden change. The convergence performance of the FDSAF and the SAF-MCC algorithm is poor. FDSAF algorithm cannot suppress a sudden change with non-Gaussian noise. The proposed FDSAF-MCC algorithm shown a convergence trend, but the convergence effect is not very good after sudden change.

### Experiment 3

In order to further verify the convergence performance under non-Gaussian noise and/or a sudden change environment, this example compares the FDSAF and FDSAF-MCC under different input signals. The input signal *x*(*n*) is generated by

6 $$\begin{aligned} x(n)=a x(n-1) + \sqrt{1-a^2}\xi (n) \end{aligned}$$

$a\in [0,1)$ is a correlation coefficient determining the correlation relation between *x*(*n*) and $x(n-1)$, and $\xi (n)$ is a white Gaussian stochastic process with zero mean and unitary variance. When $a=0$, the input signal *x*(*n*) is the white noise. When *a* is close to 1, the input signal *x*(*n*) is colored noise. In this experiment, the cases of $a = 0, 0.9$ are considered. The MSE curves of FDSAF-MCC and FDSAF are compared in the  experiment.

**Figure 10:**
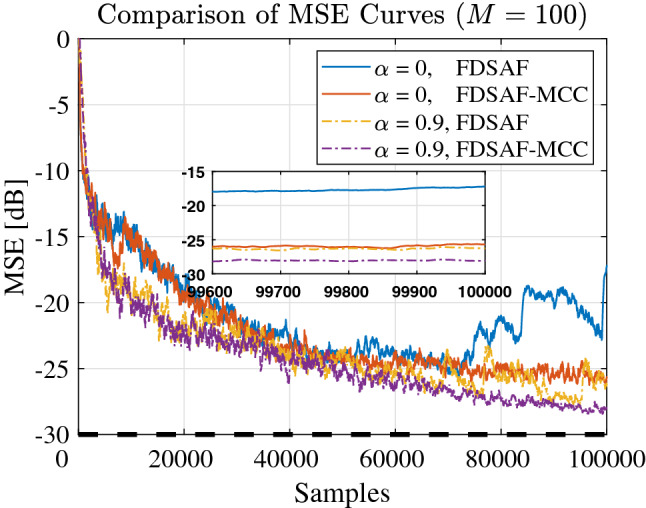
Experiment 3: MSE curves of FDSAF-MCC and FDSAF under impulsive noise with different *a*.

Figure 10 shown the MSE curves comparison between FDSAF and the proposed FDSAF-MCC under white noise input and colored noise input. The convergence performance of the FDSAF algorithm and the proposed FDSAF-MCC algorithm is compared under the same parameter *a*. When $a = 0$, input with the white noise, the proposed FDSAF-MCC algorithm has the better convergence performance than the FDSAF algorithm. When $a = 0.9$, input with the colored noise, the proposed FDSAF-MCC algorithm has the better convergence performance than the FDSAF algorithm.

**Figure 11:**
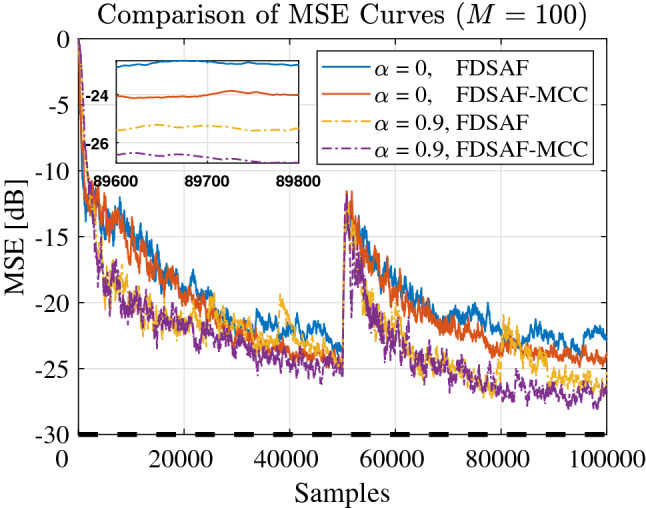
Experiment 3: MSE curves of FDSAF-MCC and FDSAF under impulsive noise and a sudden change.

Figure 11 shown the MSE curves comparison between FDSAF and the proposed FDSAF-MCC with different *a* under non-Gaussian noise and a sudden change. The convergence performance of the proposed FDSAF-MCC algorithm better than the FDSAF algorithm when in the same *a*.

## Methods

### FDSAF-MCC filtering

**Figure 12:**
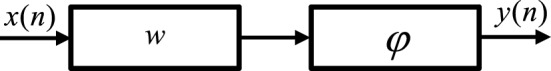
The structure of the SAF.

**Figure 13:**
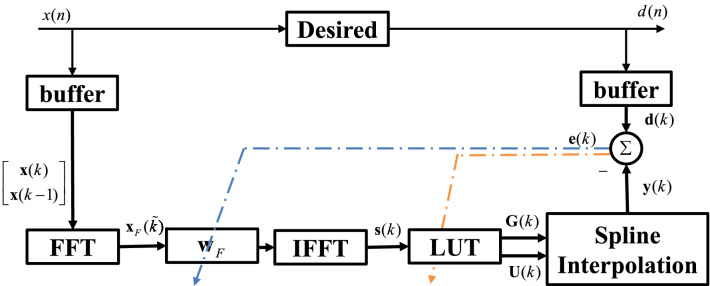
The structure of the FDSAF-MCC.

The basic structure of SAF in Fig. 12 is the cascade of a linear adaptive filter and a nonlinear cubic CR-spline interpolation function^23^. The structure of the frequency domain maximum correntropy criterion spline adaptive filter (FDSAF-MCC) is shown in Fig. 13. Frequency domain adaptive filtering (FDAF), used to linear module of the SAF^24^. During this process, the filtering and parameter updating are performed every *M* instants. Let the length of data buffer equal to the length of FIR filter weight, *M*. The input signal *x*(*n*) is stored in the data buffer ${\mathbf {x}}(k)$ for every *M* samples, where *k* denotes the data buffer index in time domain. ${\mathbf {x}}(k) = [x(kM + 1), x(kM + 2), \ldots , x(kM + M)]^T$. In order to achieve the optimal efficiency, the $50\%$ overlap-save method is used, taking the *FFT* of ${\mathbf {x}}(k)$ and ${\mathbf {x}}(k-1)$ at instant $n = kM + M$

7 $$\begin{aligned} \begin{aligned} {\mathbf {x}}_F({\tilde{k}})&=FFT[{\mathbf {x}}(k-1),{\mathbf {x}}(k)]^T\\&=FFT[x(kM-M+1),\ldots ,x(kM+M)]^T \end{aligned} \end{aligned}$$

$FFT[\cdot ]$ represents *FFT* operation for a vector, and ${\tilde{k}}$ is used to denote the index in frequency domain. The FIR filtered output ${\mathbf {s}}(k)$ can be calculated by

8 $$\begin{aligned} {\mathbf {s}}(k)= IFFT[{\mathbf {x}}_F({\tilde{k}})\odot {\mathbf {w}}_F({\tilde{k}})]^T \quad Last \ M \ elements \end{aligned}$$

${\mathbf {s}}(k)$ is containing *M* output elements from instant $n = kM + 1$ to $n = kM + M$, where $IFFT[\cdot ]$ represents *IFFT* operation for a vector. ${\mathbf {w}}_F({\tilde{k}})$ is the FIR filter weight in frequency domain. $\odot$ denotes the Hadamard product that represents matrix/vector multiplication by elements.

As shown in Fig. 13, the nonlinear spline interpolation contains table look-up and interpolation two procedures. The look-up table (LUT) is made up of $N+1$ control points (knots) defined as ${\mathbf {G}}_j=[g_{x,j},g_{y,j}]^T$, $(j=0,1,\dots ,N)$. The subscripts *x* and *y* denote abscissa and ordinate, respectively. The abscissas are uniformly distributed with an interval $\Delta x$. The LUT process will calculate the spline interval index $i_j$ and the local abscissa $u_j$ according to $s(kM+j)$ at instant $n=kM+j$,

9 $$\begin{aligned} i_j= & {} \lfloor \frac{s(kM+j)}{\Delta x} \rfloor + \frac{N-1}{2} \end{aligned}$$

10 $$\begin{aligned} u_j= & {} \frac{s(kM+j)}{\Delta x} - \lfloor \frac{s(kM+j)}{\Delta x} \rfloor \end{aligned}$$

$\lfloor \cdot \rfloor$ denotes the floor operator. Then vectorizing all normalized abscissas $u_j$ and the corresponding local spline knots $g_{i,j}$ of all $s(kM + j)$ in data buffer. ${\mathbf {u}}_j = [u_j^3 , u_j^2 , u_j , 1]^T$ is the local abscissa vector derived from $s(kM + j)$. The normalized abscissas $u_j$ are vectorized as ${\mathbf {U}}(k) \in {\mathbb {R}}^{4\times M}$ which is

11 $$\begin{aligned} {\mathbf {U}}(k) = [{\mathbf {u}}_1,{\mathbf {u}}_2,\ldots ,{\mathbf {u}}_M] \end{aligned}$$

In a similar way, $\dot{{\mathbf {u}}}_j = [3u_j^2 , 2u_j , 1 , 0]^T$ is the differential vector of a local abscissa. We denote $\dot{{\mathbf {U}}}(k) \in {\mathbb {R}}^{4\times M}$ the differential matrix of normalized abscissas, which can be expressed as

12 $$\begin{aligned} \dot{{\mathbf {U}}}(k) = [\dot{{\mathbf {u}}}_1,\dot{{\mathbf {u}}}_2,\ldots ,\dot{{\mathbf {u}}}_M] \end{aligned}$$

And we denote ${\mathbf {G}}(k) \in {\mathbb {R}}^{4\times M}$ the spline knot matrix, which can be described by,

13 $$\begin{aligned} {\mathbf {G}}(k) = [{\mathbf {g}}_{i,1},{\mathbf {g}}_{i,2},\ldots ,{\mathbf {g}}_{i,M}] \end{aligned}$$

${\mathbf {g}}_{i,j}=[g_{i,j},g_{i,j+1},g_{i,j+2},g_{i,j+3}]^T$. After FIR filtering in frequency domain, the intermediate variables *s*(*k*) will enter the spline interpolation, obtaining the output signal *y*(*k*), from instant $n=kM+1$ to $n=kM+M$, which can be written as

14 $$\begin{aligned} \begin{aligned} {\mathbf {y}}(k)&=[y(kM+1), \ldots ,y(kM+M)]^T\\&=[\varphi (s(kM+1)), \ldots ,\varphi (s(kM+M))]^T\\&=({\mathbf {U}}^T(k) \cdot {\mathbf {C}} \cdot {\mathbf {G}}(k))_{ii},i=1,2,\ldots ,M\\&= sum_r({\mathbf {U}}^T(k)\cdot {\mathbf {C}} \odot {\mathbf {G}}^T(k))\\&= sum_c({\mathbf {C}} \cdot {\mathbf {G}}(k)\odot {\mathbf {U}}(k))^T \end{aligned} \end{aligned}$$

The spline interpolation function is represented by $\varphi (\cdot )$, $y(kM + j)$ denotes the output of spline filter at instant $n = kM + j$, $(\cdot )_{ii}$ represents the column vector made up from the diagonal elements of the matrix, $sum_r(\cdot )$ represents the column vector derived from the summation of the matrix by rows, and $sum_c(\cdot )$ represents the row vector derived from the summation of the matrix by columns.

Cubic spline curves, which mainly include B-spline^25^ and Catmul-Rom (CR) spline^26^. Because of the feature that CR-spline passes through all of the control points, CR-spline may has much better performance in local approximation with respect to B-spline^27^. Therefore, CR-spline is the only one considered in the paper. The basis matrix ${\mathbf {C}}$

15 $$\begin{aligned} \begin{aligned} {{\mathbf {C}}}&= \frac{1}{2}\left[ {\begin{array}{*{20}c} {-1} &{} {3} &{} {-3} &{} {1} \\ 2 &{} {-5} &{} 4 &{} {-1} \\ {-1} &{} 0 &{} 1 &{} 0 \\ 0 &{} 2 &{} 0 &{} 0 \\ \end{array}} \right] \end{aligned} \end{aligned}$$

### FDSAF-MCC adaptive

**Figure 14:**
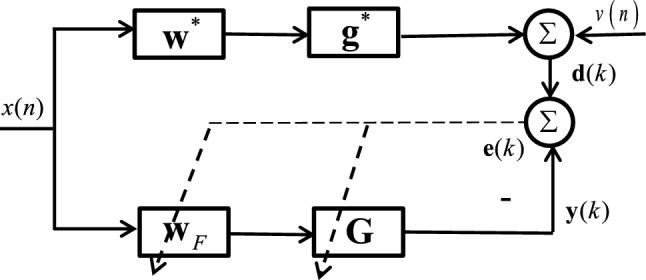
The structure of FDSAF-MCC nonlinear system identification.

As shown in Fig.14 the structure of FDSAF-MCC nonlinear system identification, we will derive the parameter updating rules. The error ${\mathbf {e}}(k)$ can be written as

16 $$\begin{aligned} \begin{aligned} {\mathbf {e}}(k)&= [e(kM+1),e(kM+2),\ldots ,e(kM+M)]^T\\&= {\mathbf {d}}(k)-{\mathbf {y}}(k) \end{aligned} \end{aligned}$$

$e(kM + j) = d(kM + j)-y(kM + j)$, at instant $n=kM+j$. ${\mathbf {d}}(k) = [d(kM + 1), d(kM + 2),\ldots , d(kM + M)]^T$ is the desired output.

An maximum correntropy cost function^19–21^ which is insensitive to impulsive noises, given by

17 $$\begin{aligned} \begin{aligned} J(k)&= \frac{1}{\sqrt{2\pi }\delta }exp(-\frac{{\mathbf {e}}^2(k)}{2\delta ^2})\\&=\frac{1}{\sqrt{2\pi }\delta }exp(-\frac{e^2(kM+j)}{2\delta ^2}) \end{aligned} \end{aligned}$$

The kernel size $\delta >0$, and $\sqrt{2\pi }\delta$ is the normalization. We take the derivative of Eq. (17) with respect to ${\mathbf {e}}(k)$, and the result described by ${\mathbf {e}}_d(k)$

18 $$\begin{aligned} \begin{aligned} {\mathbf {e}}_d(k)&=\frac{\partial J(k)}{\partial {\mathbf {e}}(k)}\\&= \frac{-1}{\sqrt{2\pi }\delta ^3}exp(-\frac{{\mathbf {e}}^2(k)}{2\delta ^2}){\mathbf {e}}(k) \end{aligned} \end{aligned}$$

${\mathbf {e}}_d(k)=[e_d(kM + 1),e_d(kM + 2),\ldots ,e_d(kM + M)]^T$. As there is no *FFT* transformation in the process of spline interpolation, so the parameter updating of spline knot is the same of time domain SAF. In order to match the data block description in FDSAF-MCC, the parameter updating can be expressed in the vectorization form. We take the derivative of *J*(*k*) with respect to ${\mathbf {g}}_{i,j}$^15^, described

19 $$\begin{aligned} \begin{aligned} \frac{\partial J(k)}{\partial {\mathbf {g}}_{i,j}}&=-e_d(kM+j){\mathbf {C}}^T{\mathbf {u}}_j \end{aligned} \end{aligned}$$

Then the derivative of *J*(*k*) with respect to ${\mathbf {G}}(k)$ can be expressed as a vectorization form

20 $$\begin{aligned} \begin{aligned} \frac{\partial J(k)}{\partial {\mathbf {G}}(k)}&=\left[ \frac{\partial J(k)}{\partial {\mathbf {g}}_{i_{1}}}, \frac{\partial J(k)}{\partial {\mathbf {g}}_{i_{2}}}, \ldots , \frac{\partial J(k)}{\partial {\mathbf {g}}_{i_{M}}}\right] \\&=-\left[ e_d(k M+1) {\mathbf {C}}^{\mathrm {T}} {\mathbf {u}}_{1}, \ldots , e_d(k M+M) {\mathbf {C}}^{\mathrm {T}} {\mathbf {u}}_{M}\right] \\&=- {\mathbf {e}}_d^{\mathrm {T}}(k) \odot {\mathbf {C}}^{\mathrm {T}} \cdot {\mathbf {U}}(k) \end{aligned} \end{aligned}$$

Therefore, the parameter updating of the spline knots matrix can be expressed as

21 $$\begin{aligned} {\mathbf {G}}(k+1) = {\mathbf {G}}(k)+\mu _g {\mathbf {e}}^T_d(k)\odot {\mathbf {C}}^{T} \cdot {\mathbf {U}}(k) \end{aligned}$$

The learning rate $\mu _g$ on the updating of ${\mathbf {G}}(k)$. We take the derivative of *J*(*k*) with respect to ${\mathbf {w}}(k)$

22 $$\begin{aligned} \begin{aligned} \frac{\partial J(k)}{\partial {\mathbf {w}}(k)}&=e_d(kM+j)\frac{\partial e(kM+j)}{\partial y(kM+j)}\frac{\partial y(kM+j)}{\partial s(kM+j)}\frac{\partial s(kM+j)}{\partial {\mathbf {w}}(k)} \end{aligned} \end{aligned}$$

In which, we denote the back propagation error $e_s (kM + j) = e_d(kM + j){\dot{\varphi }}(s(kM + j))$, then it can be vectorized as

23 $$\begin{aligned} {\mathbf {e}}_s(k)={\mathbf {e}}_d(k)\odot \dot{\mathbf {\varphi }}(k) \end{aligned}$$

${\mathbf {e}}_s (k) = [e_s (kM + 1), e_s (kM + 2),\ldots , e_s (kM + M)]^T$. The vector ${\dot{\varphi }}(k)$ reference to Eq. (14) can be rewritten as

24 $$\begin{aligned} \begin{aligned} {\dot{\varphi }}(k)&=(\dot{\,}{U}^T(k) \cdot {\mathbf {C}} \cdot {\mathbf {G}}(K))_{ii}/\Delta x, i = 1,2,\ldots ,M\\&=sum_c({\mathbf {C}} \cdot {\mathbf {G}}(K) \odot \dot{{\mathbf {U}}}(k) )^T/\Delta x \end{aligned} \end{aligned}$$

According to the procedures of FDSAF-MCC, the back propagation error vector is transformed into frequency domain by the following mean

25 $$\begin{aligned} {\mathbf {e}}_F({{\tilde{k}}}) = FFT \left[ {\begin{array}{*{20}c} {\mathbf {0}} \\ {{\mathbf {e}}_s(k)} \\ \end{array}} \right] \end{aligned}$$

A zero vector ${\mathbf {0}}\in {\mathbb {R}}^{M\times 1} = [0, 0,\ldots , 0]^T$ with length of *M*, and ${\mathbf {e}}_F({{\tilde{k}}}) \in {\mathbb {R}}^{2M\times 1}$is the back propagation error vector in frequency domain. The gradient vector $\Delta {\mathbf {w}}(k)$ be implemented in frequency domain

26 $$\begin{aligned} \Delta {\mathbf {w}}(k) = IFFT [ {\mathbf {e}}_F({{\tilde{k}}}) \odot {\mathbf {x}}^H_F({{\tilde{k}}})] \quad First \ M \ elements \end{aligned}$$

The superscript *H* represents Hermitian transpose. The length of $IFFT[{\mathbf {e}}_F({{\tilde{k}}}) \odot {\mathbf {x}}^H_F ({{\tilde{k}}})]$ is 2*M*, and the actual gradient vector is the first *M* elements of it. Finally, the parameter updating of frequency domain weight ${\mathbf {w}}_F$ achieved by

27 $$\begin{aligned} {\mathbf {w}}_F({{\tilde{k}}} + 1) = {\mathbf {w}}_F({{\tilde{k}}})+\mu _w FFT \left[ {\begin{array}{*{20}c} {\Delta {\mathbf {w}}(k)} \\ {{\mathbf {0}}} \\ \end{array}} \right] \end{aligned}$$

The learning rate $\mu _w$ on the updating of ${\mathbf {w}}_F$, and ${\mathbf {0}}\in {\mathbb {R}}^{M\times 1} = [0, 0,\ldots , 0]^T$ is a zero vector with length of *M*.

### FDSAF-MCC brief

In order to explain the FDSAF-MCC algorithm more clearly, a brief summary of FDSAF-MCC is given in Algorithmic 1. 
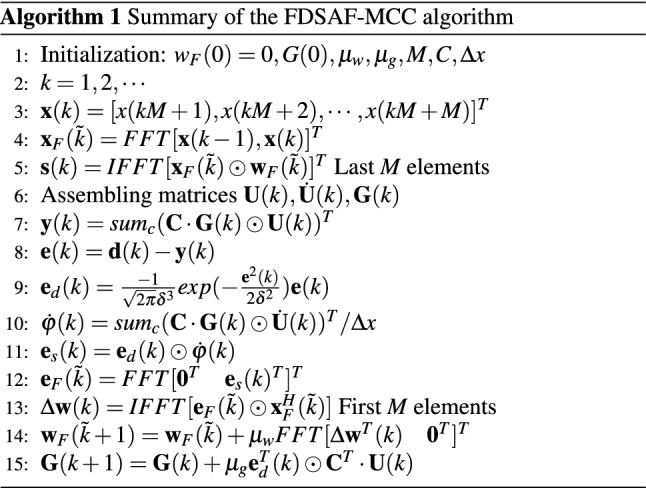


### Convergence performance

The convergence performance of the linear module and the nonlinear module are considered separately. According to the cost function in the parameter updating, $\Vert e(k+1)\Vert ^2 < \Vert e(k)\Vert ^2$ must be satisfied during filtering. We take the first order Taylor series expansion of $\Vert e(k+1)\Vert ^2$

28 $$\begin{aligned} \begin{aligned} \Vert e(k+1)\Vert ^2&= \Vert e(k)\Vert ^2 + \frac{\partial \Vert e(k)\Vert ^2}{\partial {\mathbf {G}}(k)^{\mathbf {T}}} \Delta {\mathbf {G}}(k) + h.o.t. \\&= \Vert e(k)\Vert ^2 - ({\mathbf {e}}_d^T(k)\odot {\mathbf {C}}^T \cdot {\mathbf {U}}(k))^{\mathbf {T}}(\mu _g {\mathbf {e}}_d^T(k)\odot {\mathbf {C}}^T \cdot {\mathbf {U}}(k)) + h.o.t.\\&= \Vert e(k)\Vert ^2(1 - \mu _g \Vert exp(- \frac{e^2(k)}{2\delta ^2}) {\mathbf {C}}^T {\mathbf {U}}(k) \Vert ^2) + h.o.t. \end{aligned} \end{aligned}$$

We ignore h.o.t. which represents high order terms, satisfied $\Vert e(k+1)\Vert ^2 < \Vert e(k)\Vert ^2$

29 $$\begin{aligned} 0< 1 - \mu _g \Vert exp(- \frac{e^2(k)}{2\delta ^2}) {\mathbf {C}}^T {\mathbf {U}}(k) \Vert ^2 < 1 \end{aligned}$$

The bound on learning rate $\mu _g$ for spline knots complies with

30 $$\begin{aligned} 0< \mu _g < \frac{1}{\Vert exp(- \frac{e^2(k)}{2\delta ^2}) {\mathbf {C}}^T {\mathbf {U}}(k) \Vert ^2 } \end{aligned}$$

In a similar way, we take the Taylor series expansion of $\Vert e(k+1)\Vert ^2$ about ${\mathbf {w}}(k)$, at instant *k*

31 $$\begin{aligned} \begin{aligned} \Vert e(k+1)\Vert ^2&= \Vert e(k)\Vert ^2 + \frac{\partial \Vert e(k)\Vert ^2}{\partial {\mathbf {w}}(k)^{\mathbf {T}}} \Delta {\mathbf {w}}(k) + h.o.t. \\&= \Vert e(k)\Vert ^2 - ({\mathbf {x}}_F(\tilde{k}){\mathbf {e}}_d(k)\odot \dot{\mathbf {\varphi }}(k))^{\mathbf {T}}(\mu _w{\mathbf {x}}_F(\tilde{k}){\mathbf {e}}_d(k)\odot \dot{\mathbf {\varphi }}(k)) + h.o.t.\\&= \Vert e(k)\Vert ^2(1 - \mu _w \Vert {\mathbf {x}}_F({{\tilde{k}}}) exp(- \frac{e^2(k)}{2\delta ^2}) \dot{\mathbf {\varphi }}(k) \Vert ^2) + h.o.t. \end{aligned} \end{aligned}$$

We ignore h.o.t. which represents high order terms, condition of $\Vert e(k+1)\Vert ^2 < \Vert e(k)\Vert ^2$, obtained

32 $$\begin{aligned} 0< \mu _w < \frac{1}{\Vert {\mathbf {x}}_F({{\tilde{k}}}) exp(- \frac{e^2(k)}{2\delta ^2}) \dot{\mathbf {\varphi }}(k) \Vert ^2 } \end{aligned}$$

## Conclusion

This paper proposed a new frequency domain maximum correntropy criterion spline adaptive filtering. To suppression non-Gaussian impulsive noise along with having comparable operation time. Instead of using the lest mean square (LMS), the new algorithm employed the maximum correntropy criterion (MCC) as a cost function. Three experimental methods were used to verify the effectiveness of the proposed algorithm in suppressing impulsive noise. Compared with the existing frequency domain spline adaptive filtering algorithm, the proposed algorithms provided better robustness against alpha stable noise. The proposed algorithm has a good effect in suppressing the alpha stable noise in the above-mentioned environments, but it may have a problem of poor convergence in suppressing light-tailed noise. It is necessary to further explore a more appropriate cost function and construct a filtering algorithm to suppress light-tailed models noise. In the future, for the light-tailed noise model, we will be carried out in the frequency domain spline adaptive filtering algorithm.

## References

[1] Liu C, Zhang Z (2018). Set-membership normalised least m-estimate spline adaptive filtering algorithm in impulsive noise. Electron. Lett..

[2] Liu C, Zhang Z, Tang X (2019). Sign normalised hammerstein spline adaptive filtering algorithm in an impulsive noise environment. Neural Process. Lett..

[3] Scarpiniti M, Comminiello D, Parisi R, Uncini A (2015). Novel cascade spline architectures for the identification of nonlinear systems. IEEE Trans. Circuits Syst. I Regular Papers.

[4] Scarpiniti M, Comminiello D, Scarano G, Parisi R, Uncini A (2015). Steady-state performance of spline adaptive filters. IEEE Trans. Signal Process..

[5] Liu, C., Zhang, Z. & Tang, X. Steady-state performance for the sign normalized algorithm based on hammerstein spline adaptive filtering. In *2019 International Conference on Control, Automation and Information Sciences (ICCAIS)*, 1–4 (IEEE, 2019).

[6] Guan S, Li Z (2017). Normalised spline adaptive filtering algorithm for nonlinear system identification. Neural Process. Lett..

[7] Liu C, Zhang Z, Tang X (2018). Sign normalised spline adaptive filtering algorithms against impulsive noise. Signal Process..

[8] Yang L, Liu J, Yan R, Chen X (2019). Spline adaptive filter with arctangent-momentum strategy for nonlinear system identification. Signal Process..

[9] Wen P, Zhang J, Zhang S, Qu B (2021). Normalized subband spline adaptive filter: Algorithm derivation and analysis. Circuits Syst. Signal Process..

[10] Guo, W. & Zhi, Y. Nonlinear spline adaptive filtering against non-gaussian noise. *Circuits Syst. Signal Process.* 1–18. 10.1007/s00034-021-01798-3 (2021).

[11] Yu T, Li W, Yu Y, de Lamare RC (2021). Robust spline adaptive filtering based on accelerated gradient learning: Design and performance analysis. Signal Process..

[12] Patel V, Bhattacharjee SS, George NV (2021). A family of logarithmic hyperbolic cosine spline nonlinear adaptive filters. Appl. Acoust..

[13] Kumar K, Pandey R, Bhattacharjee SS, George NV (2021). Exponential hyperbolic cosine robust adaptive filters for audio signal processing. IEEE Signal Process. Lett..

[14] Bhattacharjee SS, Kumar K, George NV (2020). Nearest kronecker product decomposition based generalized maximum correntropy and generalized hyperbolic secant robust adaptive filters. IEEE Signal Process. Lett..

[15] Yang L, Liu J, Zhang Q, Yan R, Chen X (2020). Frequency domain spline adaptive filters. Signal Process..

[16] Shi L, Lin Y (2014). Convex combination of adaptive filters under the maximum correntropy criterion in impulsive interference. IEEE Signal Process. Lett..

[17] Chen B, Xing L, Liang J, Zheng N, Principe JC (2014). Steady-state mean-square error analysis for adaptive filtering under the maximum correntropy criterion. IEEE Signal Process. Lett..

[18] Radhika, S. & Chandrasekar, A. Maximum correntropy criteria adaptive filter with adaptive step size. In *2018 IEEE International Conference on Computational Intelligence and Computing Research (ICCIC)*, 1–4 (IEEE, 2018).

[19] Peng, S., Wu, Z., Zhang, X. & Chen, B. Nonlinear spline adaptive filtering under maximum correntropy criterion. In *TENCON 2015-2015 IEEE Region 10 Conference*, 1–5 (IEEE, 2015).

[20] Wang W, Zhao H, Zeng X, Doğançay K (2019). Steady-state performance analysis of nonlinear spline adaptive filter under maximum correntropy criterion. IEEE Trans. Circuits Syst. II Express Briefs.

[21] Wu Z, Peng S, Chen B, Zhao H (2015). Robust hammerstein adaptive filtering under maximum correntropy criterion. Entropy.

[22] Weng B, Barner KE (2005). Nonlinear system identification in impulsive environments. IEEE Trans. Signal Process..

[23] Scarpiniti M, Comminiello D, Parisi R, Uncini A (2013). Nonlinear spline adaptive filtering. Signal Process..

[24] Shynk JJ (1992). Frequency-domain and multirate adaptive filtering. IEEE Signal Process. Mag..

[25] Zhao J, Zhang H, Zhang JA (2020). Generalized maximum correntropy algorithm with affine projection for robust filtering under impulsive-noise environments. Signal Process..

[26] Zhang J-F, Qiu T-S (2017). A robust correntropy based subspace tracking algorithm in impulsive noise environments. Digit. Signal Process..

[27] Comminiello D, Príncipe JC (2018). Adaptive Learning Methods for Nonlinear System Modeling.

